# Increased epicardial adipose tissue thickness is a predictor of new-onset diabetes mellitus in patients with coronary artery disease treated with high-intensity statins

**DOI:** 10.1186/s12933-017-0650-3

**Published:** 2018-01-11

**Authors:** Jeehoon Kang, Young-Chan Kim, Jin Joo Park, Sehun Kim, Si-Hyuck Kang, Young Jin Cho, Yeonyee E. Yoon, Il-Young Oh, Chang-Hwan Yoon, Jung-Won Suh, Young-Seok Cho, Tae-Jin Youn, In-Ho Chae, Dong-Ju Choi

**Affiliations:** 1 Department of Internal Medicine, Seoul National University Bundang Hospital, 82 Gumiro173 Beongil, Bundang, Seongnam, Gyeonggi 13620 South Korea; 2 Department of Internal Medicine, Seoul National University Hospital, Seoul, South Korea; 3 Division of Cardiology, Hallym University Hangang Sacred Heart Hospital, Seoul, South Korea; 4 Seoul National University College of Medicine, Seoul, South Korea

**Keywords:** Statin, New-onset diabetes mellitus, Epicardial adipose tissue, Coronary artery disease, Echocardiography

## Abstract

**Background:**

Statins are widely used for lipid lowering in patients with coronary artery disease (CAD), but increasing evidence indicates an association between statin use and new-onset of diabetes mellitus (NODM). Epicardial adipose tissue (EAT) refers to the visceral fat surrounding the heart, which is associated with metabolic diseases. We sought to determine the association between EAT thickness and NODM in CAD patients treated with high-intensity statins.

**Methods:**

We conducted a retrospective medical record review of CAD patients treated with high-intensity statins for at least 6 months after percutaneous coronary intervention performed between January 2009 and June 2013 at Seoul National University Bundang Hospital. EAT thickness was measured by echocardiography using standardized methods.

**Results:**

A total of 321 patients were enrolled, who received high-intensity statins for a mean of 952 days; atorvastatin 40 mg in 204 patients (63.6%), atorvastatin 80 mg in 57 patients (17.8%), and rosuvastatin 20 mg in 60 patients (18.7%). During the follow-up period of 3.9 ± 1.7 years, NODM occurred in 40 patients (12.5%). On Cox proportional-hazard regression analysis, EAT thickness at systole [for each 1 mm: hazard ratio (HR) 1.580; 95% confidence interval (CI) 1.346–1.854; *P* < 0.001] and prediabetes at baseline (HR 4.321; 95% CI 1.998–9.349; *P* < 0.001) were the only independent predictors of NODM. Using binary cutoff values derived from the receiver operating characteristic curve analysis, EAT thickness at systole larger than 5.0 mm had an HR of 3.402 (95% CI 1.751–6.611, *P* < 0.001), sensitivity of 52.5%, and specificity of 80.8% for predicting NODM. Also, patients with EAT thickness ≥ 5 mm and prediabetes at baseline had a 12.0-times higher risk of developing NODM compared to the risk noted in patients with EAT thickness < 5 mm and normal glucose tolerance at baseline.

**Conclusion:**

Epicardial adipose tissue thickness at systole is a consistent independent predictor of NODM in patients with CAD treated with high-intensity statins. Such predictors may help physicians plan adequate surveillance for early detection of NODM.

**Electronic supplementary material:**

The online version of this article (10.1186/s12933-017-0650-3) contains supplementary material, which is available to authorized users.

## Introduction

Epicardial adipose tissue (EAT) refers to the visceral fat surrounding the heart, which can be easily measured in the clinic with standard transthoracic echocardiography [1]. Previous studies have shown the relationship of EAT with metabolic syndrome [2], atherosclerosis [3], glucose intolerance [4] and high blood pressure [5]. Especially in patients with coronary artery disease, EAT can release free fatty acid in the proximity of coronaries arteries, which disturbs vascular homeostasis and endothelial function [6]. Based on various studies, EAT has been suggested to be a promising indicator for the detection of high cardio-metabolic risk [7].

In patients with high cardiovascular risk, statins have been widely used to lower lipid levels. Although statins are effective in reducing the rate of cardiovascular events and mortality [8], there are consistent concerns regarding the association between statin use and increased rates of diabetes mellitus (DM) [9, 10]. Despite the clinical importance of NODM in patients with cardiovascular disease, previous studies have shown inconsistent results regarding the predictors of statin-associated NODM [11–13].

Because EAT is a sensitive biomarker of metabolic status, we hypothesized that EAT thickness may be associated with the occurrence of glucose intolerance in patients with coronary artery disease (CAD) treated with high-intensity statin therapy. We also evaluated the clinical utility of EAT thickness as a predictor of NODM in these patients.

## Methods

### Study population

The study retrospectively enrolled patients who underwent percutaneous coronary intervention (PCI) between January 2009 and June 2013, received high-intensity statin treatment for at least 6 months, and had at least one baseline echocardiographic evaluation within 3 months after PCI at Seoul National University Bundang Hospital. We excluded patients with DM at baseline or no clinical/laboratory information regarding DM status, patients with a follow-up duration less than 6 months, and patients with a poor echocardiographic image quality for the measurement of EAT thickness (Additional file 1: Figure S1). PCI was performed using standard techniques, and follow-up was performed according to routine clinical guidelines. For each patient, the follow-up duration was calculated based on the prescriptions of high-intensity statins. High-intensity statin therapy, defined as either atorvastatin (40 or 80 mg) or rosuvastatin (20 or 40 mg), had been administered according to the 2013 American College of Cardiology/American Heart Association guidelines [8]. DM was defined as fasting blood glucose levels ≥ 126 mg/dL, glycated hemoglobin levels ≥ 6.5% (48 mmol/mol), and/or the need for oral hypoglycemic agents or insulin. If there was no clear clinical diagnosis, or if a patient had discordant results from two different tests, a second test was searched for confirmation. Prediabetes was defined as fasting blood glucose levels of 100–125 mg/dL or glycated hemoglobin levels of 5.7–6.4% (39–47 mmol/mol) [14].

The study protocol was approved by the Institutional Review Board of Seoul National University Bundang Hospital and was conducted according to the principles of the Declaration of Helsinki.

### Measurement of EAT thickness

All subjects underwent echocardiographic examination performed using commercially available ultrasound machines (Vivid E9, GE Healthcare, Chicago, USA; EPIQ 7, Philips Healthcare, Amsterdam, The Netherlands), and standard examination was performed with the patient in left lateral position. Left ventricular ejection fraction was calculated with the modified biplane Simpson’s method. EAT thickness was measured at the end of systole and diastole on the free wall of the right ventricle in the parasternal long-axis view on standard transthoracic echocardiography, and was defined as an echo-free or hypoechoic area adjacent to the right ventricle (Additional file 1: Figure S2). This method was validated in previous studies and shown to be strongly correlated with various metabolic markers [1, 7]. Only the maximum EAT thickness values were measured. The measurement was performed for two beats, and the average value was retained.

### Statistical analysis

Data are presented as numbers and frequencies for categorical variables and as median and interquartile ranges for continuous variables, and were compared using Student’s t-test or the Mann–Whitney U test. To compare the groups, the χ^2^ test (or the Fisher’s exact test when any expected cell count was < 5 for a 2-by-2 table) was used for categorical variables, and the unpaired Student t-test or one-way analysis of variance was applied for continuous variables.

In the multivariate analysis performed to identify variables influencing NODM, we used the multivariable Cox proportional hazard model. Candidate variables with *P* < 0.10 in the univariate analyses, duration of statin treatment, and previously described risk factors of DM [i.e., age, male sex, body mass index (BMI), and hypertension] were included in the model [15, 16]. For the sensitivity analysis of predictors of progression of glucose intolerance, we used the binary logistic model based on multiple variables. Variables included in the logistic regression model were identical to those of the multivariable Cox proportional hazard model. To determine the best cutoff value of EAT thickness that would be included in the predictive model, we performed receiver operating characteristic curve analysis. To determine intraobserver variability, one of the authors (JK) measured EAT thickness at systole and diastole twice at an interval of > 30 days. Agreement was analyzed by means of the Bland–Altman plot (Additional file 1: Figure S3) and by determination of the intraclass correlation coefficient using the two-way mixed model (coefficient for EAT thickness at systole: 0.936 [0.916–0.951]; coefficient for EAT thickness at diastole: 0.943 [0.925–0.956]).

All statistical tests were two-tailed. A two-sided probability value less than 0.05 was considered to indicate statistical significance. Statistical tests were performed using SPSS version 20 (SPSS Inc., Chicago, IL, USA).

## Results

### Baseline characteristics and EAT thickness

A total of 321 patients were enrolled in this study, according to the flow chart provided in Additional file 1: Figure S1. The mean age was 59.9 years, 74% of patients were male patients, and 64% presented with acute coronary syndrome. The patients received high-intensity statins for a median of 930 days; atorvastatin 40 mg in 204 patients (63.6%), atorvastatin 80 mg in 57 patients (17.8%), and rosuvastatin 20 mg in 60 patients (18.7%; Table 1).

**Table 1:** Baseline characteristics of the total population

	Total population	NODM (+) (n = 40)	NODM (−) (n = 281)	*P* value
Demographic findings				
Age (years)	60 (51, 69)	60 (51, 72)	59 (51, 69)	0.950
Sex (male, %)	238 (74.1%)	30 (75.0%)	208 (74.0%)	0.895
BMI (kg/m^2^)	24.9 (23.2, 27.1)	25.8 (23.7, 28.0)	24.8 (23.2, 27.0)	0.184
BMI > 25 kg/m^2^	157 (48.9%)	23 (57.5%)	134 (47.7%)	0.245
Clinical diagnosis (%)				0.984
Stable angina	115 (35.8%)	14 (35.0%)	101 (35.9%)	
Unstable angina	48 (15.0%)	7 (17.5%)	41 (14.6%)	
NSTEMI	64 (19.9%)	7 (17.5%)	57 (20.3%)	
STEMI	94 (29.3%)	12 (30.0%)	82 (29.2%)	
Hypertension (%)	127 (39.6%)	19 (47.5%)	108 (38.4%)	0.273
Current smoking (%)	84 (26.2%)	10 (25.0%)	74 (26.3%)	0.857
Previous CVA (%)	11 (3.4%)	0 (0.0%)	11 (3.9%)	0.203
Bronchial asthma (%)	5 (1.6%)	1 (2.5%)	4 (1.4%)	0.607
COPD (%)	9 (2.8%)	2 (5.0%)	7 (2.5%)	0.368
Dyslipidemia (%)	80 (24.8%)	9 (22.5%)	71 (25.3%)	0.705
Prediabetes (%)	130 (40.5%)	31 (77.5%)	99 (35.2%)	< 0.001
Laboratory findings				
WBC (/μL)	7900 (5970, 10,950)	7640 (5500, 10,450)	7900 (6000, 11,040)	0.589
Hemoglobin (g/dL)	14.6 (13.5, 15.6)	15.0 (13.6, 15.8)	14.5 (13.4, 15.6)	0.246
Fasting blood glucose (mg/dL)	92 (84, 103)	98 (90, 107)	92 (84, 102)	0.010
HbA1c (%/mmol/mol)	5.7 (5.5, 5.9)/38.8 (36.6, 41.0)	6.0 (5.7, 6.3)/42.1 (39.1, 45.4)	5.7 (5.4, 5.8)/38.8 (35.5, 39.9)	< 0.001
Total cholesterol (mg/dL)	207 (177, 240)	210 (171, 246)	207 (179, 240)	0.774
Triglyceride (mg/dL)	133 (90, 209)	145 (99, 214)	131 (89, 209)	0.414
HDL-cholesterol (mg/dL)	42 (37, 50)	40 (35, 47)	43 (37, 51)	0.100
LDL-cholesterol (mg/dL)	133 (110, 157)	126 (112, 157)	133 (108, 157)	0.979
Serum creatinine (mg/dL)	0.88 (0.73, 1.01)	0.84 (0.76-1.00)	0.98 (0.73, 1.02)	0.524
hsCRP (mg/dL)	0.15 (0.10, 0.31)	0.15 (0.10, 0.25)	0.15 (0.10, 0.32)	0.264
Echocardiography				
LVEDD (mm)	48.0 (44.3, 51.9)	49.0 (45.0, 52.1)	48.0 (44.0, 51.9)	0.408
LVESD (mm)	31.0 (27.0, 35.0)	32.0 (29.0, 34.6)	30.3 (26.9, 35.1)	0.319
LV ejection fraction (%)	60.0 (53.5, 64.7)	60.3 (54.9, 66.1)	59.5 (53.1, 64.5)	0.246
Left atrium dimension (mm)	37.1 (33.7, 41.0)	36.8 (34.1, 40.0)	37.4 (33.7, 41.0)	0.624
EAT diastole (mm)	1.4 (1.0, 2.2)	2.2 (1.4, 3.5)	1.2 (1.0, 2.1)	< 0.001
EAT systole (mm)	4.0 (3.0, 4.9)	5.4 (4.2, 7.4)	3.9 (2.9 4.8)	< 0.001
Baseline medication				
Aspirin	321 (100%)	40 (100%)	281 (100%)	NA
Clopidogrel	320 (99.7%)	40 (100%)	280 (99.6%)	0.706
ACE inhibitor or ARB	275 (85.7%)	34 (85.0%)	241 (85.8%)	0.897
Beta blockers	246 (76.6%)	30 (75.0%)	216 (76.9%)	0.794
Thiazides	36 (11.2%)	4 (10.0%)	32 (11.4%)	0.795
Systemic steroid	30 (9.3%)	3 (7.5%)	27 (9.6%)	0.668
Statin				0.128
Atorvastatin 40 mg	204 (63.6%)	20 (50.0%)	184 (65.5%)	
Atorvastatin 80 mg	57 (17.8%)	11 (27.5%)	46 (16.4%)	
Rosuvastatin 20 mg	60 (18.7%)	9 (22.5%)	51 (18.1%)	
Statin duration (days)				
Total statin duration	1248 (984, 1800)	1348 (983, 1827)	1237 (984, 1800)	0.293
High intensity statin duration	930 (541, 1216)	963 (785, 1322)	922 (500, 1210)	0.297

*ACE* angiotensin-converting enzyme, *ARB* angiotensin-receptor blocker, *BMI* body mass index, *COPD* chronic obstructive pulmonary disease, *CVA* cerebrovascular accident, *EAT* epicardial adipose tissue, *HDL* high density lipoprotein, *hsCRP* high-sensitivity C-reactive protein, *ISR* in-stent restenosis, *LDL* low density lipoprotein, *LV* left ventricular, *LVEDD* left ventricular end diastolic dimension, *LVESD* left ventricular end systolic dimension, *MI* myocardial infarction, *NSTEMI* non-ST-segment elevation myocardial infarction, *STEMI* ST-segment elevation myocardial infarction, *WBC* white blood cell

New-onset diabetes mellitus occurred in 40 patients (12.5%), with the incidence of NODM increasing gradually over the course of a mean follow-up of 3.9 years. Regarding baseline characteristics, patients with NODM had a higher frequency of baseline prediabetes, and higher levels of fasting blood glucose and HbA1c. Furthermore, among echocardiographic variables, EAT thickness at diastole and systole were significantly larger in the NODM group than in the non-NODM group (Additional file 1: Figure S4), whereas other variables did not show significant difference between the two groups. Regarding the relationship between variables, we found a moderate positive correlation of EAT thickness with HbA1c (Pearson correlation coefficient 0.307, P < 0.001, Additional file 1: Figure S5).

### Predictors of NODM

**Fig. 1:**
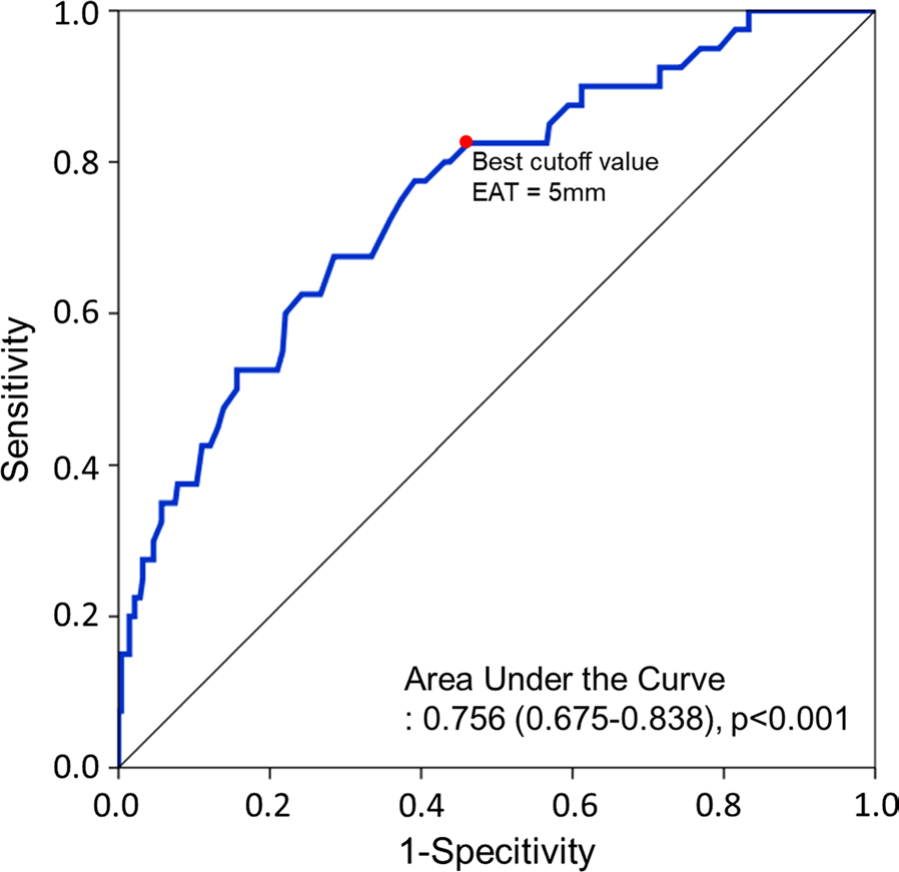
The receiver operating characteristic curve for EAT thickness at systole and corresponding area under the curve (AUC) statistics for the risk of NODM

Regarding factors associated with NODM, univariate Cox regression analysis showed that EAT thickness and prediabetes at baseline were significant predictors of NODM (Table 2), which remained significant after multivariate adjustment for significant covariates (for each 1 mm of EAT thickness at systole: hazard ratio (HR) of 1.580, 95% confidential interval (CI) of 1.346–1.854, *P* < 0.001; for prediabetes at baseline: HR 4.321, 95% CI 1.998–9.349, *P* < 0.001; Table 2). Using binary cutoff values derived from the receiver operating characteristic curve analysis, EAT thickness at systole equal to or larger than 5.0 mm showed a sensitivity of 52.5%, and specificity of 80.8% for predicting NODM (Fig. 1). Moreover, the HR for NODM in patients with EAT thickness ≥ 5 mm was 3.402 (95% CI 1.751–6.611, *P* < 0.001), showing that EAT thickness remained as an independent predictor of NODM after adjusting clinical variables, including prediabetes at baseline.

**Table 2:** Univaraite and multivariate analyses for new-onset diabetes mellitus

Factor	Univariate analysis		Multivariate analysis	
	HR (95% CI)*	*P* value	HR (95% CI)*	*P* value
Age	0.999 (0.975–1.023)	0.909	0.978 (0.950–1.007)	0.130
Male sex	1.029 (0.502–2.108)	0.938	1.220 (0.539–2.765)	0.633
BMI	1.062 (0.956–1.179)	0.263	0.996 (0.877–1.131)	0.996
Diagnosis as acute coronary syndrome	0.954 (0.497–1.830)	0.888	–	–
Hypertension	1.524 (0.818–2.838)	0.184	1.727 (0.872–3.420)	0.117
Current smoking	1.030 (0.503–2.109)	0.935	–	–
Dyslipidemia	1.114 (0.530–2.341)	0.775	–	–
Prediabetes at baseline	5.503 (2.619–11.564)	< 0.001	4.321 (1.998–9.349)	< 0.001
Anemia (Hemoglobin < 12 g/dL)	2.745 (0.377–19.985)	0.319	–	–
TG level (per mg/dL)	1.001 (0.999–1.004)	0.309	–	–
LDL-cholesterol level (per mg/dL)	1.002 (0.993–1.011)	0.703	–	–
LV ejection fraction < 40%	2.153 (0.296–15.672)	0.449	–	–
EAT thickness at diastole (per mm)	1.625 (1.353–1.950)	< 0.001		
EAT thickness at systole (per mm)	1.611 (1.388–1.870)	< 0.001	1.580 (1.346–1.854)	< 0.001
Total statin duration (per year)	0.916 (0.716–1.171)	0.482	0.876 (0.733–1.048)	0.147
High intensity statin duration	0.989 (0.804–1.217)	0.918	–	–

*BMI* body mass index, LV left ventricle, *LDL* low density lipoprotein, *TG* triglyceride, *EAT* epicardial adipose tissue

* The hazard ratio (HR) along with its corresponding 95% confidence interval (CI) and p values are based on Cox proportional hazard analysis

### Risk factors of NODM

**Fig. 2:**
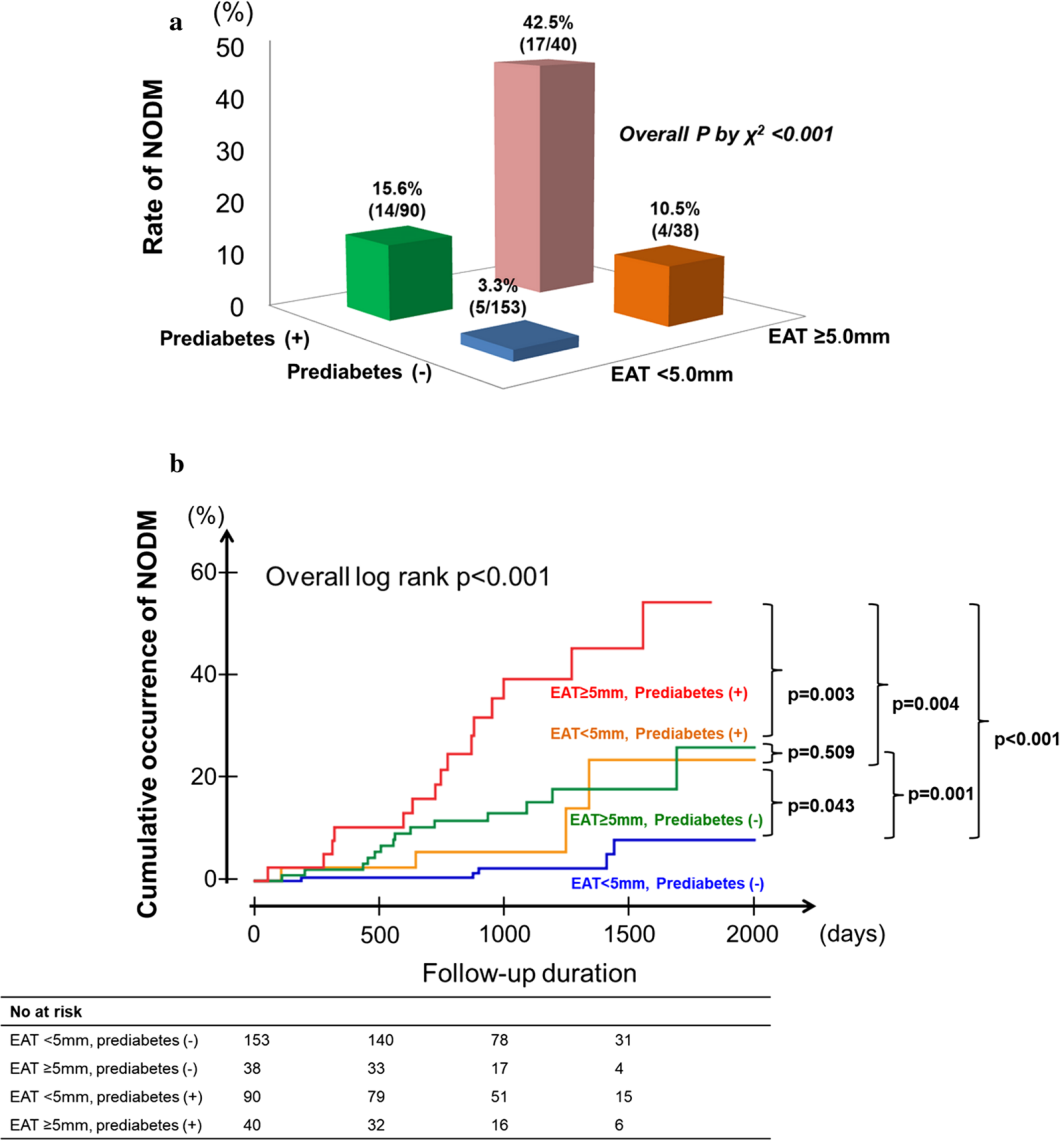
Incidence of new-onset diabetes mellitus (NODM) according to epicardial adipose tissue (EAT) thickness at systole and the presence of prediabetes. **a** Among the total population, 153 patients had an EAT < 5 mm with no prediabetes at baseline (Group 1), 38 patients had an EAT ≥ 5 mm with no prediabetes at baseline (Group 2), 90 patients had an EAT < 5 mm with prediabetes at baseline (Group 3), and 40 patients had an EAT ≥ 5 mm with prediabetes at baseline (Group 4). Patients with EAT ≥ 5.0 mm and prediabetes at baseline had the highest incidence of NODM. Post-hoc analysis of NODM incidence showed that there were significant differences between all pairs of groups, except between Group 2 and Group 3 (Group 1 vs. Group 2, *P* = 0.012; Group 1 vs. Group 3, *P* = 0.001; Group 1 vs. Group 4, *P* < 0.001; Group 2 vs. Group 3, *P* = 0.661; Group 2 vs. Group 4, *P* < 0.001; Group 3 vs. Group 4, *P* < 0.001). **b** Kaplan–Meier survival curve showing an incremental increase in risk for NODM, according to the presence of prediabetes at baseline and EAT thickness

When we stratified the patients into four groups according to EAT thickness at systole and presence of prediabetes, the incidence of NODM was highest in patients with EAT thickness ≥ 5 mm and prediabetes at baseline (17 out of 40 patients; 42.5%), which was 12.0-fold higher than that in patients with EAT thickness < 5 mm and without prediabetes at baseline (5 out of 153 patients; 3.3%, Fig. 2a). On Kaplan–Meier curve analysis and Cox regression analysis, patients with both risk factors had a significantly higher risk for NODM (Fig. 2b, Table 3). Meanwhile, patients with either one of the risk factors (i.e., those with EAT thickness ≥ 5 mm without prediabetes and those with EAT thickness < 5 mm with prediabetes) had a similar risk for NODM (*P* = 0.509).

**Table 3:** Risk of new-onset diabetes mellitus according to the epicardial adipose tissue thickness and prediabetes

	HR	95% CI	*P* value
EAT thickness < 5 mm and no prediabetes	Reference		
EAT thickness ≥ 5 mm and no prediabetes	3.481	0.934–12.972	0.063
EAT thickness < 5 mm and prediabetes	5.011	1.805–13.916	0.002
EAT thickness ≥ 5 mm and prediabetes	14.702	5.336–40.503	< 0.001

*EAT* epicardial adipose tissue, *HR* hazard ratio, *CI* confidence interval

### Sensitivity analysis for progression of glucose intolerance

To evaluate the association between EAT thickness and gradual impairment in glucose tolerance, we stratified the patients into two groups according to the progression of glucose intolerance (progression vs. no progression; Additional file 2: Table S1). Progression of glucose intolerance was noted in about 40% of the total study population (progression group), among whom 81 patients (25.2%) showed normal glucose tolerance at baseline and developed new-onset prediabetes during the follow-up period. The progression group had more prediabetic patients, higher levels of fasting blood glucose, and marginally longer statin treatment duration. Additionally, analysis of echocardiographic findings revealed larger EAT thickness at systole in the progression group (4.2 [3.2, 5.4] vs. 3.8 [2.9, 4.8] mm, *P* = 0.014; Additional file 2: Table S2). A logistic regression model including age, sex, BMI, hypertension, statin duration, prediabetes, and EAT thickness at systole showed that EAT thickness was an independent predictor of progression of impaired glucose tolerance (for each 1 mm of EAT thickness at systole: odds ratio of 1.309, 95% CI 1.117–1.534, *P* = 0.001), and so was prediabetes (odds ratio of 3.265, 95% CI 1.919–5.555, *P* < 0.001; Additional file 2: Table S3).

## Discussion

In this study involving patients who received PCI and were prescribed high-intensity statins for at least 6 months, NODM occurred in 12.5% of patients during a follow-up period of 3.9 years. Baseline EAT thickness at systole and prediabetes at baseline were revealed as two independent predictors for NODM. Patients with EAT thickness ≥ 5 mm and prediabetes at baseline had a 12.0-fold higher risk to develop NODM compared to the risk noted in patients without risk factors. Considering that echocardiography is performed in nearly all CAD patients undergoing PCI, our study provides an easy-to-obtain predictor of NODM in patients who require high-dose statin treatment.

### Statins and risk of NODM in cardiovascular disease

Statins are effective therapeutic agents for prevention of cardiovascular events, and can reduce mortality in patients with coronary heart disease [17]. However, recent studies reported that statin treatment may be associated with an increased risk of NODM. A meta-analysis of 13 trials involving 91,140 individuals showed that statin treatment was associated with a 9% increase in the 4-year risk of NODM [10]. Regarding the dose–effect relationship in statin-associated NODM, some studies have shown a higher risk of incident diabetes in patients on higher-intensity statin therapy [9, 18], while some suggest that there might be difference in the incident diabetes by statin class [19]. Large scale studies focusing on the occurrence of NODM by statin intensity or by statin class should be conducted to give us clear answers on this issue. Our present study found a similar rate of NODM in patients using high-intensity statins during 4 years of follow-up. Regarding previous reports showing that NODM is associated with a substantial risk for mortality [20], further efforts should be allocated to the early prediction and prevention of NODM [21].

### Biological relevance of the EAT

The EAT is known as the true visceral fat deposit of the heart, lying directly on the epicardial surface of the myocardium within the pericardial sac [22]. Due to its close proximity to the coronary vessels, the EAT exerts profound effect on the local physiology of the myocardium and the coronary vasculature by expressing various cytokines [23]. Additionally, EAT was associated with cardiovascular risks and further development of cardiovascular complications [24]. EAT thickness has also been shown to be related to the metabolic status of the individual. Specifically, Yorgun et al. reported that EAT thickness was significantly increased in patients with metabolic syndrome, and that age and BMI, which are factors related to metabolic syndrome, were the strongest independent predictors of EAT thickness [25]. A recent meta-analysis also showed that EAT thickness was significantly higher in patients with metabolic syndrome [26]. Other studies have explained that the association between metabolic syndrome and EAT thickness may be attributed to the endocrine action of the EAT, which also affects insulin sensitivity [27], designating EAT as a biologically active organ. Conversely, some studies suggested the beneficial effect of EAT by protecting the heart against myocardial stress, hypertension, and local inflammation. EAT may even function as a brown adipose tissue store which can protect adjacent tissues from hypothermia, while showing high degrees of white adipose tissue lipolysis allowing the buffering of high toxic levels of free fatty acids [6]. Additionally, a genetic study explored the EAT transcriptome, unveiling a majority of genes involved in coagulation, endothelial function, phospholipase activity, apoptosis, and immune signaling [28]. Despite these beneficial effects, EAT may shift from being protective to detrimental for obesity and cardiovascular homeostasis [6]. Although the mechanisms that regulate the balance between protective and harmful effects of EAT are not clearly understood, epicardial fat can serve as target for pharmaceutical agents targeting the adipose tissue [29]. Furthermore, the association of EAT and diabetes has been studied in a few studies. Increased EAT thickness was independently associated with the prevalence of diabetes, insulin resistance and cardiac contractile dysfunction in diabetes [30, 31]. In the present study, we found that EAT thickness was closely associated with NODM. Aside from prediabetes, which is a well-known risk factor for DM, EAT thickness was the only other significant predictor of statin-associated NODM.

### Clinical implications of EAT thickness as a predictor of statin-associated NODM

Previous studies have reported conflicting results regarding the potential predictors of NODM. Specifically, the IDEAL study suggested that only patients who already have elevated risk for DM are at increased risk to develop statin-associated DM [32]. Furthermore, the Justification for Use of Statins in Prevention: an Intervention Trial Evaluating Rosuvastatin (JUPITER) study reported that the risk of statin-associated NODM was independent of baseline glucose levels, whereas Waters et al. reported that the development of NODM can be predicted based on baseline fasting glucose levels and other components of the metabolic syndrome (i.e., triglyceride levels, BMI, and hypertension) [33]. On the other hand, a cohort-based study by Woestijne et al. found that the increase in the risk of type 2 DM with statin therapy was independent of metabolic syndrome or insulin resistance [13]. The discrepancies in these previous observations may be attributed to the differences in the study population, as well as to variable statin dosage and duration.

In the present study, we limited our study population to patients with CAD who underwent PCI and required strict lipid-lowering therapy and we were able to draw a conclusion based on a relatively homogeneous small sample of patients. Moreover, considering that echocardiography is performed in nearly all CAD patients receiving PCI, our finding that EAT thickness is a predictor of statin-associated NODM may have considerable clinical implication.

Despite the risk of statin-associated NODM, the general consensus is that the positive effects of statins outweigh the negative effects on metabolic control [34]. The TNT study showed that patients at risk of statin-associated NODM also obtained substantial benefit from high-intensity statins [11]. This finding may be related to the traditional effect of statins, which lower blood cholesterol levels, and have favorable pleiotropic effects on endothelial function, oxidative stress and inflammation [35]. Nevertheless, in clinical practice, it remains important to identify factors that can estimate the risk of statin-associated NODM.

### Limitations

Several limitations should be noted. The study population was relatively small compared to cohorts investigated in previous studies or randomized controlled trials. Furthermore, we may have introduced selection bias by excluding patients prescribed with high-intensity statins for less than 6 months and those only prescribed with low- or moderate-intensity statin. Moreover, the patients in our study had various patterns of statin usage; specifically, some were not statin-naïve, and had been prescribed low- or moderate-intensity statins before receiving high-intensity statins. We also used a single method to measure EAT thickness. A previous study proposed a method to measure EAT thickness at the anterior interventricular groove, which may be more accurate than our method [36]. However, this measurement was not applicable in our retrospective analysis, because this echocardiographic view was not routinely used in our institute. Furthermore, our study did not include a control arm, which made it impossible to investigate the association between high-dose statin and NODM. However, this was not the purpose of our study, but rather its background. Therefore, the findings of our investigation may be considered as hypothesis-generating, and further large-scale studies are warranted.

## Additional files

**Additional file 1: Figure S1.** Selection of Study population. **Figure S2.** Method of EAT thickness measurement. Representative figure of EAT measurement. EAT thickness was measured at the end of systole and diastole at the free wall of the right ventricle, in the parasternal long axis view. **Figure S3.** Bland–Altman plot for Intra-observer variability. A Bland–Altman plot proved excellent agreement between the two measurements of EAT thickness at systole within one observer. **Figure S4.** A scatter plot of EAT thickness and the occurrence of new-onset diabetes mellitus. **Figure S5.** Linear correlation between HbA1c and EAT thickness.

**Additional file 2: Table S1.** Changes in glucose tolerance status. **Table S2.** Baseline clinical characteristics of the total population, grouped by progression of glucose intolerance. **Table S3.** Multivariate analysis for progression in impairment of glucose tolerance.

## Abbreviations

DM: diabetes mellitus
NODM: new-onset diabetes mellitus
EAT: epicardial adipose tissue
CAD: coronary artery disease
PCI: percutaneous coronary intervention
HR: hazard ratio
CI: confidential interval
