# Supplementary figures and images for: Increased epicardial adipose tissue thickness is a predictor of new-onset diabetes mellitus in patients with coronary artery disease treated with high-intensity statins
Source: Cardiovasc Diabetol. 2018 Jan 11;17:10. doi: 10.1186/s12933-017-0650-3 (PMC5763639; doi:10.1186/s12933-017-0650-3)

Figure S1

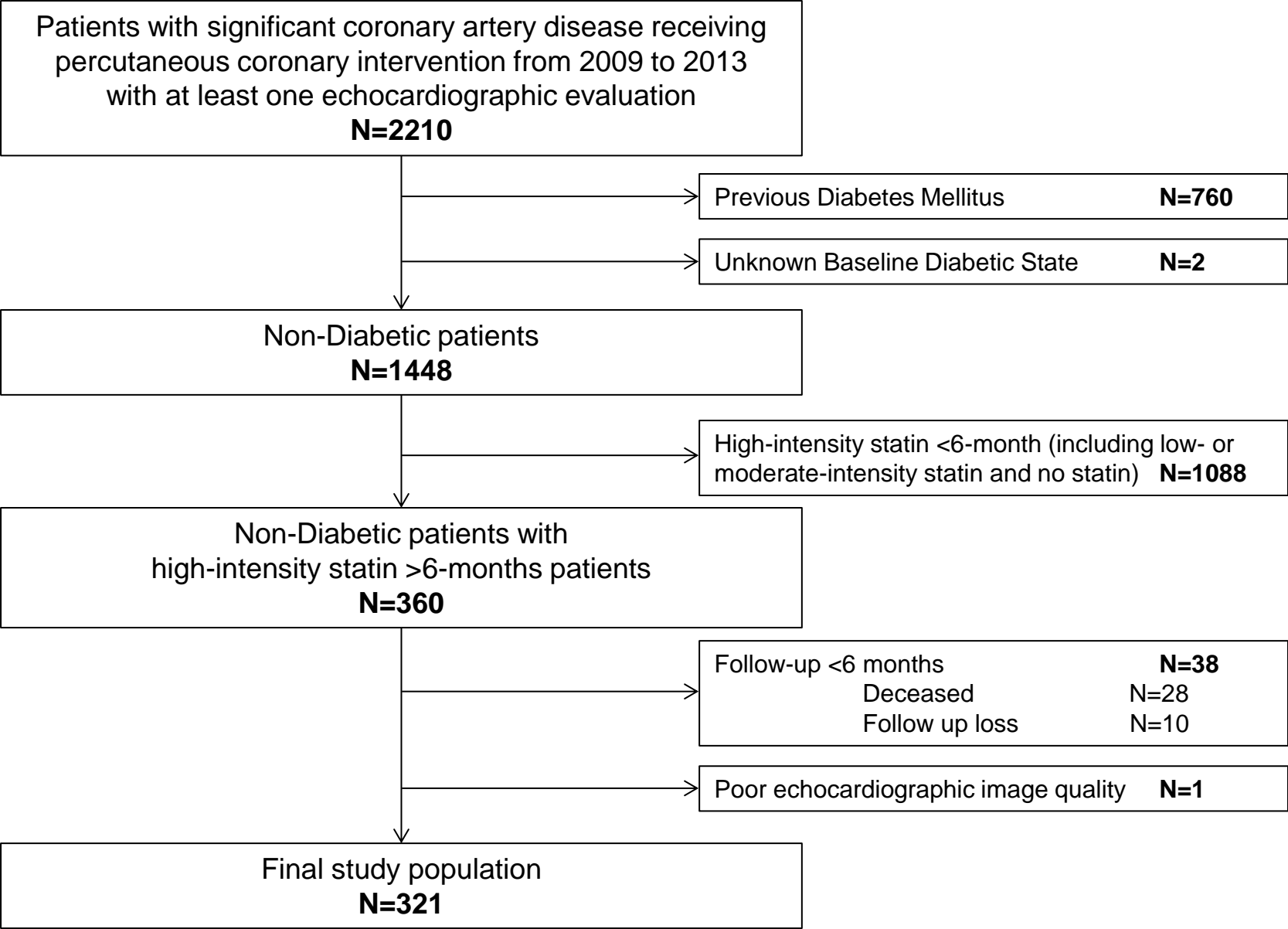

Figure S2

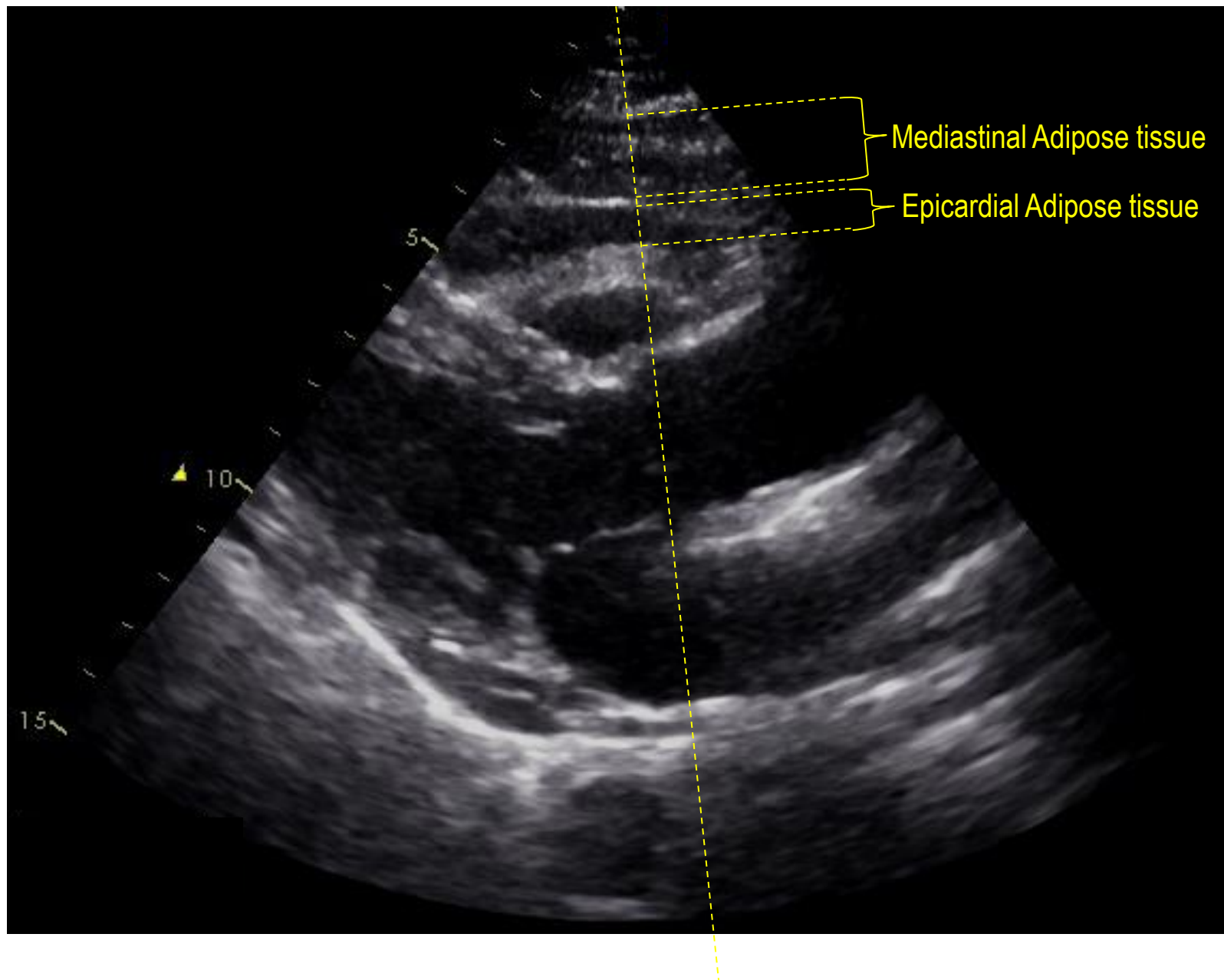

Figure S3

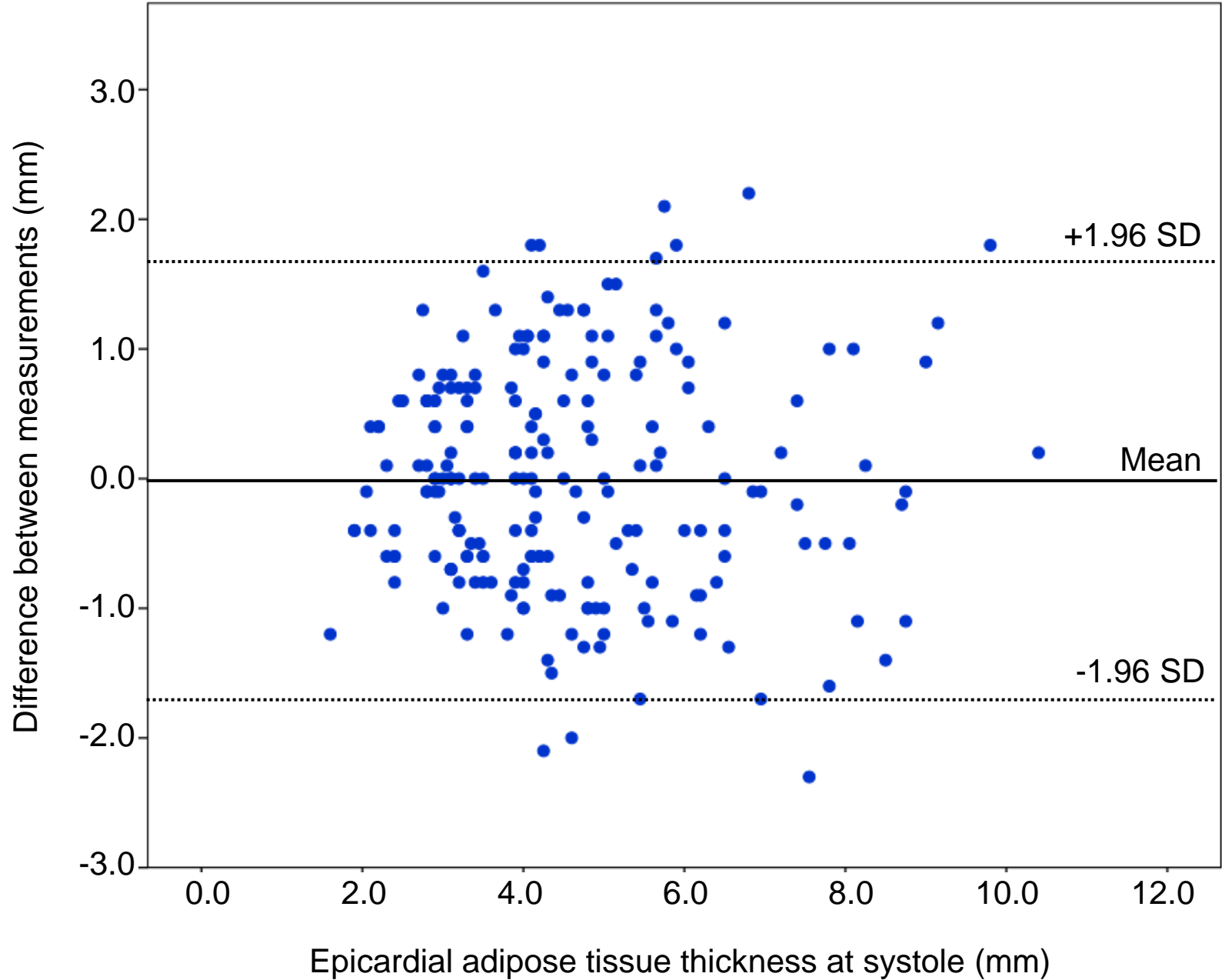

Figure S4

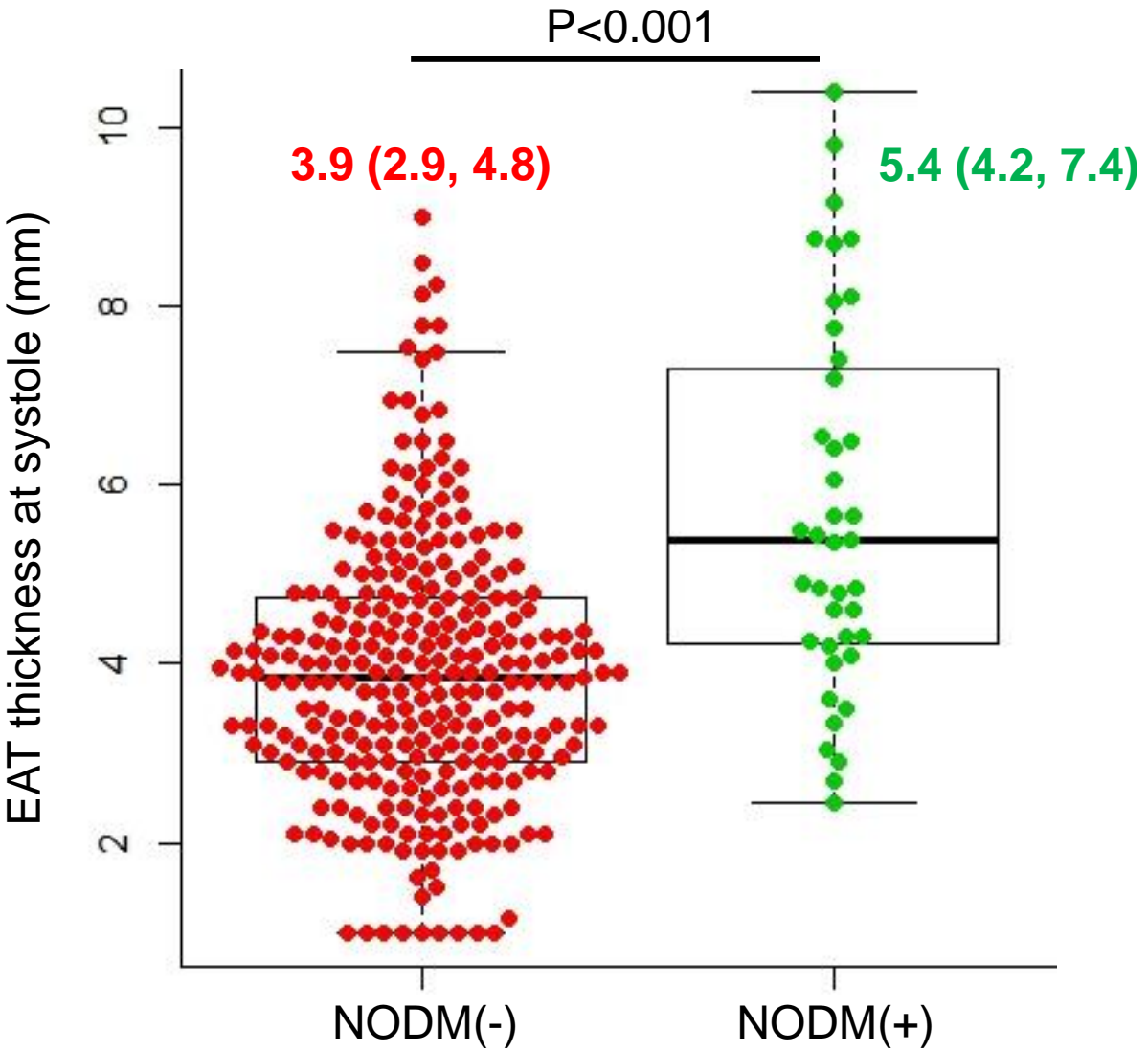

Figure S5

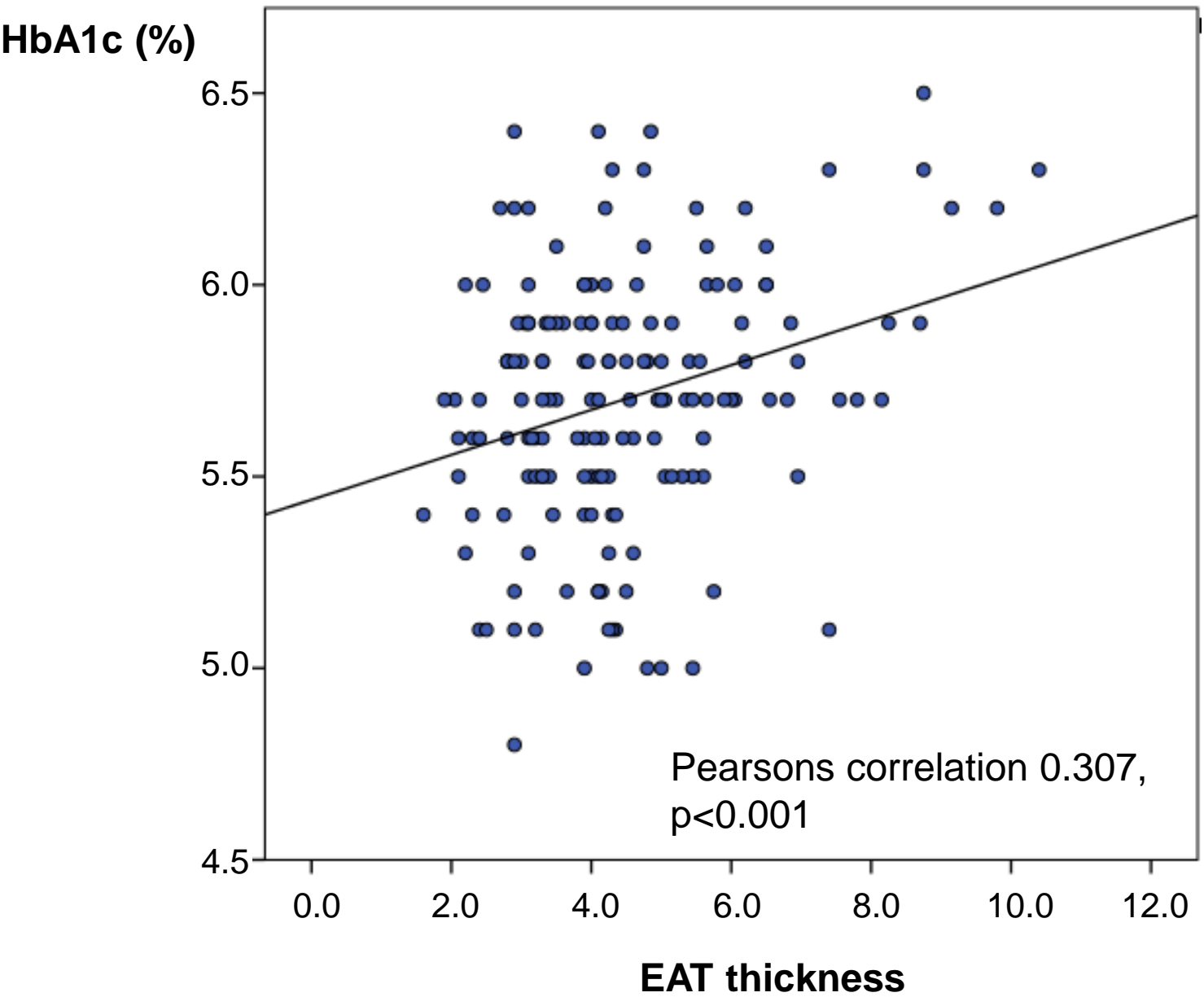

Supplement: Supplementary file 1 — Additional file 1: Figure S1. Selection of Study population. Figure S2. Method of EAT thickness measurement. Representative figure of EAT measurement. EAT thickness was measured at the end of systole and diastole at the free wall of the right ventricle, in the parasternal long axis view. Figure S3. Bland–Altman plot for Intra-observer variability. A Bland–Altman plot proved excellent agreement between the two measurements of EAT thickness at systole within one observer. Figure S4. A scatter plot of EAT thickness and the occurrence of new-onset diabetes mellitus. Figure S5. Linear correlation between HbA1c and EAT thickness. [file 12933_2017_650_MOESM1_ESM.pdf]
