# Supplementary material for: Increased epicardial adipose tissue thickness is a predictor of new-onset diabetes mellitus in patients with coronary artery disease treated with high-intensity statins
Source: Cardiovasc Diabetol. 2018 Jan 11;17:10. doi: 10.1186/s12933-017-0650-3 (PMC5763639; doi:10.1186/s12933-017-0650-3)
Supplement: Supplementary file 2 — Additional file 2: Table S1. Changes in glucose tolerance status. Table S2. Baseline clinical characteristics of the total population, grouped by progression of glucose intolerance. Table S3. Multivariate analysis for progression in impairment of glucose tolerance. [file 12933_2017_650_MOESM2_ESM.docx]

**Appendix**

**Increased Epicardial Adipose Tissue Thickness is a Predictor of**

**New-Onset Diabetes Mellitus in Patients on High-Intensity Statin Therapy**

**after Percutaneous Coronary Intervention**

Jeehoon Kang et al.

**1. Additional file Table**

**2. Additional file** Figures and Figure legends

**1. Additional file Table**

**Table S1. Changes in glucose tolerance status**

| Glucose tolerance status | | Group | N (%) |
| --- | --- | --- | --- |
| Baseline | **At Follow-up** |  |  |
| Normal glucose tolerance | Normal glucose tolerance | No Progression | 101 (31.5%) |
| Prediabetes | Prediabetes |  | 97 (30.2%) |
| Normal glucose tolerance | New-onset prediabetes | Progression | 81 (25.2%) |
| Normal glucose tolerance | New-onset Diabetes Mellitus |  | 9 (2.8%) |
| Prediabetes | New-onset Diabetes Mellitus |  | 33 (10.3%) |

**Table S2. Baseline clinical characteristics of the total population, grouped by progression of glucose intolerance**

|  | Progression (n=123) | No progression (n=198) | P value |
| --- | --- | --- | --- |
| **Demographic findings** |  |  |  |
| Age (years) | 59.2±11.4 | 60.4±13.5 | 0.412 |
| Sex (male ratio, %) | 89 (72.4%) | 149 (75.3%) | 0.565 |
| BMI (kg/m^2^) | 25.3±2.9 | 25.1±3.1 | 0.622 |
| BMI > 25 kg/m^2^ | 62 (50.4%) | 95 (48.0%) | 0.672 |
| Clinical diagnosis (%) |  |  | 0.412 |
| Stable angina | 43 (34.9%) | 72 (36.3%) |  |
| Unstable angina | 17 (13.8%) | 31 (15.7%) |  |
| NSTEMI | 31 (25.2%) | 33 (16.7%) |  |
| STEMI | 32 (26.0%) | 62 (31.3%) |  |
| Hypertension (%) | 50 (40.7%) | 77 (38.9%) | 0.754 |
| Current Smoking (%) | 33 (26.8%) | 51 (25.8%) | 0.832 |
| Bronchial Asthma (%) | 1 (0.8%) | 4 (2.0%) | 0.396 |
| Previous CVA (%) | 9 (4.5%) | 2 (1.6%) | 0.215 |
| COPD (%) | 4 (3.3%) | 5 (2.5%) | 0.701 |
| Dyslipidemia (%) | 31 (25.2%) | 49 (24.7%) | 0.927 |
| Prediabetes (%) | 97 (49.0%) | 33 (26.8%) | <0.001 |
| **Laboratory findings** |  |  |  |
| WBC (/μL) | 8900±3870 | 8710±3410 | 0.645 |
| Hemoglobin (g/dL) | 14.7±1.7 | 14.4±1.6 | 0.112 |
| Fasting blood glucose (mg/dl) | 94.2±12.1 | 91.1±13.4 | 0.049 |
| HbA1c (% / mmol/mol) | 5.8±0.6 / 39.4±6.5 | 5.7±0.3 / 38.5±3.7 | 0.185 |
| Total cholesterol (mg/dl) | 213±49 | 209±44 | 0.517 |
| Triglyceride (mg/dl) | 173±125 | 158±103 | 0.237 |
| HDL-cholesterol (mg/dl) | 44±11 | 44±9 | 0.570 |
| LDL-cholesterol (mg/dl) | 133±38 | 132±35 | 0.820 |
| Serum creatinine (mg/dl) | 0.89±0.35 | 0.89±0.22 | 0.946 |
| hsCRP (mg/dl) | 0.42±1.23 | 0.62±1.67 | 0.275 |
| **Echocardiography** |  |  |  |
| LVEDD (mm) | 47.9±4.9 | 48.1±6.0 | 0.697 |
| LVESD (mm) | 30.8±5.7 | 32.1±7.1 | 0.071 |
| LV ejection fraction (%) | 59.2±8.5 | 57.5±9.8 | 0.109 |
| Left atrium dimension (mm) | 36.9±5.1 | 37.8±6.6 | 0.178 |
| EAT diastole (mm) | 1.9±1.1 | 1.7±1.1 | 0.245 |
| EAT systole (mm) | 4.4±1.8 | 3.9±1.6 | 0.010 |
| **Baseline medication** |  |  |  |
| Aspirin | 123 (100%) | 198 (100%) | NA |
| Clopidogrel | 123 (100%) | 197 (99.5%) | 0.430 |
| ACE inhibitor or ARB | 105 (85.4%) | 170 (85.9%) | 0.903 |
| Beta blockers | 96 (78.0%) | 150 (75.8%) | 0.637 |
| Thiazides | 14 (11.4%) | 22 (11.1%) | 0.940 |
| Systemic steroid | 11 (8.9%) | 19 (9.6%) | 0.845 |
| Statin |  |  | 0.938 |
| Atorvastatin 40mg | 77 (62.6%) | 127 (64.1%) |  |
| Atorvastatin 80mg | 23 (18.7%) | 34 (17.2%) |  |
| Rosuvastatin 20mg | 23 (18.7%) | 37 (18.7%) |  |
| Statin duration (days) |  |  |  |
| Total statin duration | 1230±511 | 1114±512 | 0.050 |
| High intensity statin duration | 974±512 | 940±532 | 0.570 |

ACE, angiotensin-converting enzyme; ARB, angiotensin-receptor blocker; BMI, body mass index; COPD, chronic obstructive pulmonary disease; CVA, cerebrovascular accident; EAT, epicardial adipose tissue; HDL, high density lipoprotein; hsCRP, high-sensitivity C-reactive protein; ISR, in-stent restenosis; LDL, low density lipoprotein; LV, left ventricular; LVEDD, left ventricular end diastolic dimension; LVESD, left ventricular end systolic dimension; MI, myocardial infarction; NSTEMI, non-ST-segment elevation myocardial infarction; STEMI, ST-segment elevation myocardial infarction; WBC, white blood cell

**Table S3. Multivariate analysis for progression in impairment of glucose tolerance**

| Factor | HR | 95% CI | P |
| --- | --- | --- | --- |
| Age | 0.986 | 0.965 – 1.008 | 0.207 |
| Male sex | 1.068 | 0.597 – 1.910 | 0.824 |
| BMI | 1.019 | 0.935 – 1.109 | 0.673 |
| Hypertension | 1.040 | 0.623 – 1.736 | 0.880 |
| Prediabetes at baseline | 3.265 | 1.919 – 5.555 | <0.001 |
| EAT thickness at systole (per mm) | 1.309 | 1.117 – 1.534 | 0.001 |
| Total statin duration (per year) | 1.149 | 0.967 – 1.366 | 0.114 |

BMI, body mass index; EAT, epicardial adipose tissue
